# Validity of the DEXA diagnosis of involutional osteoporosis in patients with femoral neck fractures

**DOI:** 10.4103/0019-5413.58609

**Published:** 2010

**Authors:** Ali Humadi, Rajit H Alhadithi, Sabhan I Alkudiari

**Affiliations:** Department of Orthopaedic, Surgical Specialization Hospital, Baghdad Teaching Medical City, Baghdad, Iraq

**Keywords:** DEXA, femoral neck fracture, osteoporosis

## Abstract

**Background:**

There exists no study comparing dual energy X-ray absorptimetry (DEXA) with histomorphometry to evaluate its accuracy and validity as an assessment tool. A prospective study was done comparing the measurements of osteoporosis in patients with femoral neck fractures using the histological method of diagnosis and in the same patients with DEXA postoperatively.

**Patients and Methods:**

The histological method depends on histomorphometric analysis of bone biopsies taken from the neck of femur during surgical treatment of the fracture. We depend on three indices in histomorphometric analysis: these are osteoid seam width, osteoblast surface, and osteoid surface. The radiological method depends on the measurement of the bone mineral density using DEXA for fractured patients with the scan performed onto the contralateral nonfractured hips and lumbar spines.

**Results:**

We found positive histological histomorphometric parameters of osteoporosis in 68% of patients with the femoral neck fracture, and there is a moderate correlation between histological histomorphometric analysis and DEXA in the diagnosis of osteoporosis in these patients. In our study, DEXA can detect up to 88.2% of possible cases of osteoporosis (sensitivity 88.2%), but the specificity of this diagnostic tool is 62.5% at a t-score of ≤ −2, i.e., it is sensitive but less specific. The mean difference in the t-score in femoral DEXA and lumbar DEXA is almost zero.

**Conclusions:**

DEXA is a noninvasive and an affordable and easy method for the diagnosis of osteoporosis but less efficient than the histological histomorphometric method of diagnosis with a low specificity. We also found that the mean difference in the t-score in femoral DEXA and lumbar DEXA is almost zero, so DEXA of one region can reflect the change in the other region and there is no need for DEXA of both regions as a routine unless indicated for a special reason. This avoids exposing the patient to unnecessary risk of radiation and reduces cost.

## INTRODUCTION

Osteoporosis is the most common generalized disease of the skeleton. It causes reduction in the bone mass and change in the bone structure, both of which eventually result in reduced bone strength and increased propensity to fractures.^1^

Osteoporosis may be assessed quantitatively by radiography, densitometry studies, or histological methods^2^ and is associated with progressive weakening of bones^3^ and an increased incidence of fractures.^4^

The measurement of the trabecular bone volume in iliac crest biopsies permits the early diagnosis of osteoporosis.^3^ Histomorphometry is the gold standard for assessing bones and the degree of osteoprosis because it is the only method for the direct analysis of bone cells, bone mattress, and their activities.^5^

Bone mineral density (BMD) measurement by dual energy X-ray absorptiometry (DEXA) is widely regarded as the most important determinant of bone fragility, strength, and fracture risk.^6^

With the introduction of noninvasive radiographic techniques to measure the bone density, osteoporosis may be defined clinically as a mass per unit volume of a normally mineralized bone that falls below a population-defined threshold for a spontaneous fracture.^7^ This structural weakness of a bone is associated with a loss of trabecular bone volume, enlargement of medullary space, cortical porosity, and reduction in cortical thickness.^8^ This reduction in bone mass is associated with an increased risk of fractures, which in turn results in pain and deformity.

Till date, there is no prospective study comparing the DEXA as a widely used screening method to histomorphometry as a gold standard in the diagnosis of involutional osteoporosis in humans. Such study can give us a good idea about the accuracy and the validity of DEXA as an assessment tool. A prospective study was done comparing the measurements of osteoporosis in patients with a femoral neck fracture using the histological method of diagnosis and in the same patients with DEXA postoperatively.

## PATIENTS AND METHODS

In a prospective cross-sectional study from June 2001 to January 2005, 75 patients with fresh femoral neck fractures were studied. There were 56 females (75%) and 19 males (25%); their ages ranged from 48 to 82 years. Cases with a history of disease that may result in secondary osteoporosis such as thyrotoxicosis, steroid or heparin therapy, excessive alcohol intake, or heavy smoking were excluded. X-ray evidence of lumbosacral spondylotic changes were also excluded as lumbar osteophytes give false results in the DEXA examination.

**Figure 1:**
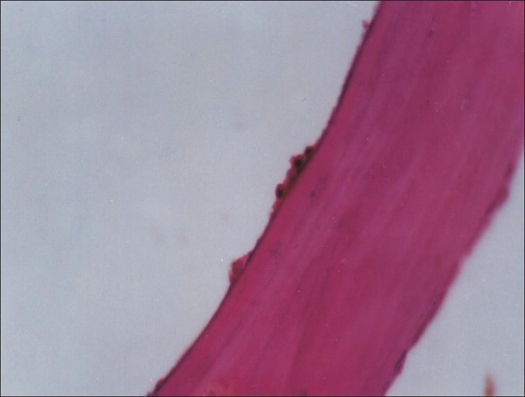
Histomorphometric slide showed clearly dark osteoid seam covering the surface with scattered osteoblasts. OST 13μm, O.S 25% and OBS 12% (non osteoporotic). Magnification 10×40×0.8×0.8

Bone biopsies were obtained from 75 femoral necks during the operative treatment of femoral neck fractures. Two biopsies (each 1 cm × 1 cm × 0.5 cm) were taken from each patient. Each biopsy specimen was put in 10% formalin and sent for histopathological slide preparation and slides were histomorphometrically studied under a high-power field [Figure 1] and the following were measured:

1. Osteoid seam width (OSW) (width of the osteoid-seam-lined bone trabecula). Three measurements were taken from each slide and the average width was taken.
2. Osteoblast surface (OBS) (fraction of the trabecular bone lined by osteoblasts). Three measurements were taken from each slide and the average width was taken.
3. Osteoid surface (OS) (fraction of the trabecular bone lined by the osteoid seams).

The average of the three readings was taken. The slides were studied.

The same patients were sent for DEXA of the contralateral hip and lumbar spine 5–10 days after the operation. BMD was measured for the lumbar spine in the anteroposterior (A-P) view at regions L1 to L4. The nonfractured hip region was scanned in the A-P position with the patient in a supine position and patient's lower leg to be examined was internally rotated.

## RESULTS

The results presented were based on the analysis of 75 patients with femoral neck fractures undergoing hip replacement. These patients gave their consent to participate in the study, and a bone tissue sample was taken for the histological evaluation of bone density. The age ranged between 48 and 82 years with a mean of 57.8 ± 11.8 (±SD). One patient included in this study with age of 48 because she developed fracture dislocation of the hip (fracture though the neck and femoral head cephalic to the fovea and she had bipolar prosthesis). Males constituted 25% of the sample. The male-to-female ratio was 1:3. Falls with an indirect impact on the hip such as sideways, backward and straight down (low energy trauma) formed 92.2% of patients while RTA (high energy trauma) constituted for only 7.8%. Those patients with falls as the mechanism of injury can be divided into two main groups according to the situation of patients during falls. A total of 16.6% patients fell from a seated or lying position on a hard surface and 83.4% fell while walking mainly indoors.

### Histological histomorphometric parameters for the diagnosis of osteoporosis

Three histomorphometric parameters were evaluated for the cases. These were the OBS, OS (presented as percentage), and finally the OSW.

These three parameters had a preset cutoff values for defining a porotic bone (histological evidence of osteoporosis for the specific parameter). An osteoid seam width < 8.8 *μ*m was considered a positive evidence of osteoporosis.^9^ The remaining two parameters were considered as positive for osteoporosis if the specimen scored < 20%.^9^ Since it is shown in Table 1 that some of the cases had only one positive histological parameter (6.7%), 22.7% had two positive criteria, and 45.3% had all three positive criteria, and to make use of all the information available from the three parameters, a reasonably specific histological definition of osteoporosis was used. Patients were considered to have a diagnosis of osteoporosis if they had at least two out of three positive histological evidence of osteoporosis; such a definition is called the gold standard for the diagnosis of osteoporosis.^9^

**Table 1:** The prevalence of osteoporosis defined on histological basis

	Cases	
	
	N	%
Histological evidence of osteoporosis		
Osteoid seam width (average thickness of the osteoid seam) < 8.8 μm	45	60
Osteoid surface (fraction of the trabecular bone lined by osteoid seams) < 20%	46	61.3
Osteoblast surface (fraction of the trabecular bone lined by osteoblasts) < 20%	50	66.7
Positive histological evidence of osteoporosis (a combination of two or three positive criteria above)	51	68
Count of histological evidence of osteoporosis		
0	19	25.3
1	5	6.7
2	17	22.7
3	34	45.3
Total	75	100
Median	2	

**Figure 2:**
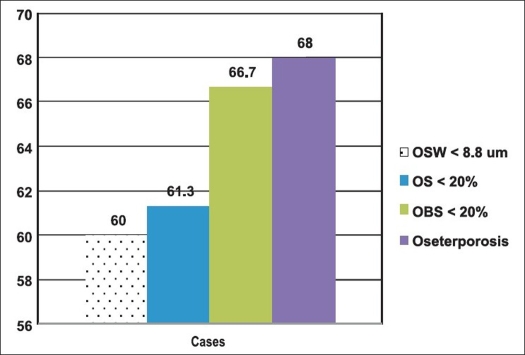
Bar chart comparing the prevalence rate of osteoporosis defined by three histological indices

As shown in Figure 2, among cases with a femoral neck fracture the prevalence of positive histological evidence of osteoporosis ranged from as low as 60% for the osteoid seam width to as high as 66.7% for the osteoblast surface. Using the previously mentioned operational definition of osteoporosis, the prevalence among cases was significantly high (68%).

### Evaluation of bone density by DEXA

The bone density of cases with a femoral neck fracture was also evaluated by DEXA. Because DEXA instrumentation is provided by several different manufacturers, the output varies in absolute terms. Consequently, it has become a standard practice to relate the results to “normal” values using t-scores, which compare individual results to the mean in a young population that is matched for race and gender and measures its difference in terms of SD units. According to the WHO definition, the *t*-score of –1 to –2.5 is defined as osteopenia and that less than or equal to 2.5 is defined as osteoporosis.

**Figure 3:**
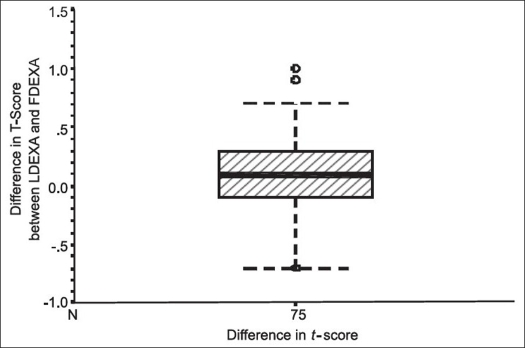
Box plot showing the distribution of differences in t-score between FDEXA and LDEXA

**Figure 4:**
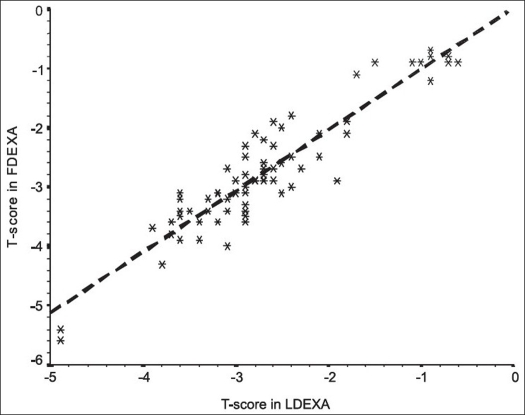
Scatter diagram (with fitted regression line) showing the correlation between the t-scores in FDEXA and LDEXA

In the present study, two body areas were examined by DEXA, the lumbar vertebrae (LDEXA) and the femoral neck (FDEXA). No statistically significant differences were observed in t-scores measured in the two areas. The mean difference in the t-score between LDEXA and FDEXA was almost zero and fluctuated equally in both positive and negative directions (random variation) [Figure 3]. Finally, there was a statistically significant strong positive linear correlation between the t-score measured in the lumbar area and that measured in the femoral neck [Figure 4]. It seemed therefore logical to use the results of the t-score in FDEXA to represent the radiological evaluation of bone density.

As shown in Table 2, the prevalence of osteoporosis defined by the t-score in both femoral neck and lumbar spine areas was comparable (72–73.3%).

**Table 2:** Distribution of cases with femoral neck fractures by *t*-score results in LDEXA and FDEXA

	N	%
*t*-score in FDEXA		
Normal (–1 to 1)	9	12
Osteopenia (–1 to –2.5)	12	16
Osteoporosis (≤–2.5)	54	72
Total	75	100
*t*-score in LDEXA		
Normal (–1 to 1)	7	9.3
Osteopenia (–1 to –2.5)	13	17.3
Osteoporosis (≤–2.5)	55	73.3
Total	75	100

### Validity of FDEXA in diagnosing osteoporosis

As shown in Table 3, FDEXA was used as a decision tool in the final diagnosis of osteoporosis defined using the gold standard definition of osteoporosis. Two preselected cutoff values for the t-score were used to define positive radiological criteria of osteoporosis: a more sensitive one employing a low cutoff value (≤–1) and a more specific one employing a higher cutoff value (≤–2.5). Using the lower cutoff value of ≤–1, DEXA would be 94.1% sensitive in detecting possible cases with osteoporosis, i.e., can be used as a screening tool in such a low cutoff value. However, a high proportion of false +ve cases would be included (75%) because of a low specificity (25%). The positive predictive value (PPV) would be low (72.7%), i.e., a positive result would give an accurate diagnosis of osteoporosis with 72.7% confidence only. The value of DEXA at this cutoff value in establishing the diagnosis of osteoporosis would be low since in such a sample with a femoral neck fracture in which one has a high index of suspicion that the patient is osteoporotic (two-thirds of the present sample has histological evidence of osteoporosis), a PPV of 72.7% would imply no obvious contribution to the diagnosis based on clinical judgment alone. At the other extreme, the NPV is also low (66.7%), i.e., a negative result would exclude the presence of osteoporosis with 66.7% confidence only.

**Table 3:** The validity parameters of t-score measurements in FDEXA in the diagnosis of osteoporosis defined on histological basis

	Final diagnosis of osteoporosis based on histology				
		
	Negative	Positive	Total	
t-score in FDEXA indicative of osteoporosis (<–1)				Sensitivity	= 94.1
Negative	6	3	9	Specificity	= 25
Positive	18	48	66	PPV	= 72.7
Total	24	51	75	NPV	= 66.7
				False +ve	= 75
				False −ve	= 5.9
				Accuracy	= 72
t-score in FDEXA indicative of osteoporosis (<–2.5)				Sensitivity	= 88.2
Negative	15	6	21	Specificity	= 62.5
Positive	9	45	54	PPV	= 83.3
Total	24	51	75	NPV	= 71.4
				False +ve	= 37.5
				False −ve	= 11.8
				Accuracy	= 80

Using the WHO cutoff value for the definition of osteoporosis based on the *t*-score of ≤ –2.5 the specificity is increased from 25% to 62.5%. This increase in the specificity is still inadequate to establish the diagnosis of osteoporosis with a reasonably high confidence since the PPV is only 83.3%. The sensitivity is lower (88.2%), i.e., DEXA at this cutoff value can detect up to 88.2% of possible cases with osteoporosis.

The sensitivity of 94.1% for the DEXA t-score at the low cutoff value of ≤ –1 and the specificity (62.5%) or PPV (83.3%) at the high cutoff value of ≤ –2.5 would be useful indicators for the physician in deciding treatment for osteoporosis in addition to other parameters derived from patient history and clinical examination.

Final diagnosis of osteoporosis based on histology was defined as a combination of two or all three positive indices of osteoporosis on histopathology; these are (1) osteoid seam width (average thickness of the osteoid seam *<* 8.8 μm), (2) osteoid surface (fraction of the trabecular bone lined by osteoid seams < 20%), and (3) osteoblast surface (fraction of the trabecular bone lined by osteoblasts < 20%).

## DISCUSSION

Postmenopausal osteoporosis affects females within 10–20 years after menopause.^10^ Vertebral fractures, femoral neck fracture, and Colle's fracture are the main clinical presentations; however, in age-related osteoporosis that affects elderly males and females above 70 years, femoral neck and vertebral fractures are the main clinical presentation.^11^

In our study, 68% of patients with femoral neck fractures showed two or three positive histomorphometric histological parameters for osteoporosis. This indicates that osteoporosis is a very important risk factor in femoral neck fractures [Table 1 and Figure 2].

Hordon and Peacock^12^ showed osteoporosis in 35% and Levenets and Pohodaieva^13^ in 75% of patients with femoral neck fractures by a histological method. This may be due to differences in the histological definition of osteoporosis. This may be attributed to the different criteria used in the diagnosis of osteoporosis or due to international variation in the incidence of osteoporosis in different countries due to differences in the standard of living and cultural issues.

DEXA is the main screening tool available in the diagnosis and management of osteoporosis in most medical practice. This study was designed to validate and compare this noninvasive tool to a more invasive but direct method of assessment of bone quality, i.e., histomorphometry.

This study showed a moderate correlation between DEXA and histomorphometry in the assessment and diagnosis of osteoporosis in hip fracture patients [Table 3]. There is no previous study to the best of the knowledge of the author comparing DEXA with histomorphometry using human samples as taking Iliac bone biopsy is invasive and nonjustifiable. Monteagudo in 1997^14^ found that there was a significant difference between histomorphometry and BMD measurement by DEXA and he also questioned this discrepancy. Wu^15^ in his assessment of BMD in postmenopausal women with and without vertebral fractures using CT scan and DEXA found that CT scan was better in the assessment.

There are several factors that can affect the specificity of DEXA adversely as a screening tool. This low specificity may be attributed to:

1. Inclusion criteria because we considered patients with osteopenia (t-score –1 to –2.5) as normal (nonosteoporotic) and in the histological method we consider patients with one positive histomorphometric index of osteoporosis as nonosteoporotic.
2. errors in measurements of BMD by DEXA.

### Most common errors

#### A. Errors in the hip region scan

1. The analysis box is misplaced: most femoral neck analysis box placement errors resulted from insufficient attention to the exact location of the box's inferolateral corner, which should have been placed immediately adjacent to the greater trochanter.^16^,^17^
2. Misshapen analysis region: the analysis region which should be rectangular is misshapen.^16^,^17^

#### B. Errors in the lumbar spine scan

1. Misplaced intervertebral disc space markers: The proper localization of disc space margins proved difficult, and this is a serious source of error, because of obscured visualization resulting from the obliquity caused by the normal lordosis of the lumbar spine.^18^ A cushion can be placed beneath the legs of all patients to minimize the lordosis, but this maneuver is not always effective. To correct the errors, the disc space markers are placed in their most logical positions, taking into account the spinal contour and using the visible positions of the posterior elements as a guide.^16^
2. Mislabeled vertebrae: Major errors result from the misidentification of the rib-bearing T12 vertebra, since all the vertebrae are thereby mislabeled. This error, however, is most easily correctable by a person familiar with the normal anatomy and its variations, including the occasional riblets that articulate with L1, as well as the occasional absence of ribs on T12.^16^,^19^
3. Artifacts analyzed as bone: The most severe error occurred when radiopaque artifacts within the region of interest were erroneously analyzed as bone. Aortic calcifications superimposed over the spine cause only an insubstantial increase in the calculated BMD.^20^

On the other hand, histomorphometry is invasive, time consuming, and subjected to interobserver errors; these together make it unsuitable for screening and picking up patients with osteoporosis. Histomorphometry is still the most reliable definitive method of diagnosis of osteoporosis and should be considered for all atypical cases of osteoporosis or when DEXA results do not fit the clinical diagnosis.

Another finding in this study is that the mean difference in the t-scores between LDEXA and FDEXA was almost zero. This small difference was not significant statistically [Figures 2 and 3]. Our results do not concur with Felix *et al*.^21^ They found that there is a difference in the DEXA studies of femoral neck and lumbar spine (the femoral neck is more osteoporotic than the lumbar spine in the same group of patients). This finding is keeping with most other results.^4^,^8^ Because involutional osteoporosis is a generalized disease and affects all bones of the body and fractures occur in certain sites more than other because the stress in these sites is higher.^22^

In fractured hip patients, DEXA showed osteoporosis (t-score –2.5) in 72% by FDEXA and 73.3% by LDEXA while osteopenia (t-score –1 to –2.5) was found in 16% by FDEXA and 17.3% by LDEXA [Table 2]. These results keep in line with Levenets (1997) who stated that 84% of patients with femoral neck fractures had either mild or severe osteoporosis.

## CONCLUSION

DEXA is a noninvasive and an affordable and easy method for the diagnosis of osteoporosis but less efficient than the histological histomorphometric method of diagnosis with low specificity as shown in this prospective study. Further evaluation of DEXA in comparison with bone histomorphometry is required in different age groups, which is impractical using human samples as histopathology is an invasive procedure. Animal sample study is a good alternative.

We also found that the mean difference in the t-scores in FDEXA and LDEXA is almost zero, so DEXA of one region can reflect the change in the other region and there is no need for DEXA of both regions as a routine unless indicated for a special reason. This avoids exposing the patient to unnecessary risks of radiation and reduces cost.
